# Metabolic immune checkpoints in cancer: how tumor-derived metabolites shape immunotherapy resistance

**DOI:** 10.3389/fimmu.2026.1894658

**Published:** 2026-07-23

**Authors:** Renjie Pan, Dongdong Chen, Yilu Wu, Lancheng Zhang, Jing Zhang, Junjie Li, Qiuxia Zhao, Yan Tang

**Affiliations:** 1 Department of Clinical Laboratory, Xinghua People’s Hospital Affiliated to Yangzhou University, Xinghua, Jiangsu, China; 2 Department of Clinical Laboratory, The Fifth People’s Hospital of Ganzhou, Ganzhou, Jiangxi, China; 3 Department of Critical Care Medicine, The Third People’s Hospital of Longgang District Shenzhen, Shenzhen, Guangdong, China; 4 Xinghua People's Hospital Affiliated to Yangzhou University, Xinghua, Jiangsu, China

**Keywords:** adenosine, immunotherapy resistance, lactate, metabolic immune checkpoint, tryptophan metabolism, tumor microenvironment

## Abstract

Immune checkpoint blockade has transformed cancer therapy, yet many tumors remain intrinsically resistant or acquire resistance after initial response. Increasing evidence indicates that this failure is not determined solely by PD-1, PD-L1, CTLA-4, or T-cell exhaustion, but also by metabolically suppressive states within the tumor microenvironment. Tumor-derived metabolites can function as metabolic immune checkpoints by limiting effector immune activity, promoting regulatory or myeloid suppressive compartments, and weakening immunotherapy efficacy. This mini review summarizes recent experimental evidence showing how lactate, adenosine, tryptophan-derived metabolites, and nucleotide-derived metabolites shape immune escape and resistance to immune checkpoint blockade. Lactate links tumor glycolysis to Treg recruitment, impaired T-cell function, and lactylation-associated therapeutic resistance. The CD73-adenosine axis suppresses CD8^+^ T cells and natural killer cells while reinforcing regulatory and myeloid immune programs. Tryptophan-derived metabolites extend beyond the classical IDO1–kynurenine–AhR pathway to involve non-classical checkpoints such as Siglec-15 and broader kynurenine/indole/serotonin networks. Emerging evidence further identifies nucleotide-derived UDP signaling as a driver of macrophage-mediated immunosuppression. Finally, we discuss how targeting metabolic checkpoints in combination with immune checkpoint blockade may improve therapeutic responses. Defining the spatial and cellular contexts of metabolite-mediated immune suppression may enable more precise strategies to overcome immunotherapy resistance.

## Highlights

- Tumor-derived metabolites can act as functional immune checkpoints that suppress antitumor immunity beyond PD-1/PD-L1 signaling.
- Lactate, adenosine, tryptophan-derived metabolites, and nucleotide-derived UDP signals regulate Tregs, TAMs, CD8^+^ T cells, NK cells, and myeloid suppressive niches.
- Combining metabolic checkpoint blockade with immune checkpoint blockade may provide new strategies to overcome immunotherapy resistance.

## 1 Introduction

Immune checkpoint blockade (ICB) has reshaped the therapeutic landscape of multiple malignancies by restoring antitumor T-cell activity through targeting inhibitory receptors or ligands such as programmed cell death protein 1 (PD-1), programmed death ligand 1 (PD-L1), and cytotoxic T-lymphocyte-associated protein 4 (CTLA-4) (1–3). These therapies have produced durable clinical responses in subsets of patients and have established immune escape as a therapeutically actionable hallmark of cancer. However, the overall efficacy of ICB remains limited in many tumor types (4–6). A substantial proportion of patients either fail to respond initially or develop acquired resistance after transient benefit. Importantly, resistance cannot always be explained by the absence of tumor-infiltrating lymphocytes, low PD-L1 expression, or insufficient immune recognition. In some tumors, CD8^+^ T cells are present but functionally impaired; in others, immune cells are spatially excluded, metabolically exhausted, or surrounded by suppressive stromal and myeloid compartments (7–9). These observations suggest that immune resistance is not governed solely by receptor–ligand checkpoint interactions, but also by the broader ecological state of the tumor microenvironment.

One important but often underappreciated layer of this ecology is metabolic suppression. Tumor cells undergo profound metabolic reprogramming to support rapid proliferation, survival under stress, and adaptation to nutrient-poor or hypoxic environments. In doing so, they consume essential nutrients, release immunomodulatory metabolites, acidify the extracellular space, and reshape the functional state of surrounding immune and stromal cells. Unlike normal immune activation, which requires coordinated metabolic switching to support effector functions, antitumor immune cells within tumors often encounter glucose deprivation, lactate accumulation, hypoxia, adenosine enrichment, altered amino acid availability, and lipid or nucleotide-derived suppressive signals (10–12). These metabolic pressures can impair T-cell receptor signaling, cytokine production, cytotoxicity, mitochondrial fitness, antigen presentation, and immune cell trafficking. Therefore, the metabolic landscape of the tumor microenvironment can act as a powerful determinant of whether antitumor immunity is sustained or suppressed.

In this context, the concept of metabolic immune checkpoints has emerged. Metabolic immune checkpoints can be broadly defined as tumor- or microenvironment-derived metabolites, metabolic enzymes, or metabolite-sensing pathways that restrict antitumor immune responses in a manner analogous to classical immune checkpoints. However, unlike PD-1 or CTLA-4, which mainly operate through defined receptor–ligand interactions on immune cells, metabolic checkpoints act through both extracellular and intracellular mechanisms. They may function as soluble signaling molecules, enzymatically generated suppressive products, nutrient-depletion programs, receptor-mediated metabolic cues, or post-translational modifications. Their targets are also diverse, including CD8^+^ T cells, regulatory T cells (Tregs), tumor-associated macrophages (TAMs), neutrophils, myeloid-derived suppressor cells, natural killer cells, dendritic cells, and cancer-associated fibroblasts (13–15). This broad range of actions allows metabolic checkpoints to reinforce immune escape at multiple levels, including T-cell dysfunction, Treg recruitment, myeloid polarization, stromal remodeling, and resistance to immunotherapy. To clarify the conceptual boundary between classical and metabolic immune checkpoints, classical immune checkpoints are defined here as receptor–ligand inhibitory pathways, such as PD-1/PD-L1 and CTLA-4/CD80-CD86, that directly restrain immune-cell activation through immune synapse-associated signaling (16). By contrast, metabolic immune checkpoints refer to metabolite-centered suppressive mechanisms that limit antitumor immunity by altering nutrient availability, extracellular metabolite accumulation, metabolic enzyme activity, metabolite-sensing receptor signaling, post-translational modification, or myeloid-cell metabolic education (17, 18). Based on their dominant mode of action, these checkpoints can be grouped into soluble metabolite checkpoints, such as lactate, kynurenine, and UDP-related nucleotide signals; metabolic enzyme checkpoints, such as LDHA, IDO1/TDO, CD39, and CD73; metabolite-sensing receptor checkpoints, such as GPR81/HCAR1, A2A/A2B receptors, AhR, and UDP-responsive purinergic receptors; metabolic post-translational modification checkpoints, represented by lactylation; and myeloid cell-mediated metabolic checkpoints, in which tumor-derived metabolites reprogram TAMs, MDSCs, or neutrophil-associated inflammatory circuits (19–21). This classification separates metabolic checkpoints from classical receptor-centered checkpoints while explaining how both systems cooperate to produce immunotherapy resistance.

Several tumor-derived metabolites have become particularly relevant to this framework. Lactate, once viewed mainly as a glycolytic waste product, is now recognized as an active immunoregulatory signal that can promote Treg recruitment, suppress effector T-cell function, and induce protein lactylation. Adenosine, generated through ectonucleotidases such as CD39 and CD73, suppresses T-cell and natural killer cell activity while supporting immunosuppressive myeloid and regulatory compartments (22–24). Tryptophan-derived metabolites, including kynurenine and related branches, link amino acid metabolism to immune escape through pathways such as AhR and emerging non-classical checkpoint axes. More recently, nucleotide-derived metabolites have also been implicated in macrophage-mediated immunosuppression and resistance to ICB, expanding the landscape of metabolic immune regulation beyond the classical lactate–adenosine–tryptophan triad.

Recent experimental studies have provided direct evidence supporting these concepts. Lactate has been shown to promote immune resistance through GPR81-dependent regulation of CX3CL1 and Treg recruitment in highly glycolytic gastric cancer, while ACVR2A attenuation in hepatocellular carcinoma links hyperglycolysis, lactate production, Treg attraction, and reduced PD-1 blockade sensitivity (25). Elevated protein lactylation has further been associated with an immunosuppressive microenvironment and therapeutic resistance in pancreatic ductal adenocarcinoma (26). In parallel, CD73-mediated adenosine production has been shown to limit antitumor immunity and enhance resistance to anti-PD-1 therapy in intrahepatic cholangiocarcinoma, and neutrophil extracellular traps can promote hepatocellular carcinoma immune escape by upregulating CD73 through Notch2 (27). Tryptophan metabolism has also been connected to immune evasion through a kynurenine/Siglec-15 axis in head and neck squamous cell carcinoma, while pharmacological reprogramming of kynurenine/indole and serotonin branches can restore ICB response in ovarian cancer (28). Finally, cancer cell nucleotide metabolism has been shown to fuel UDP-driven macrophage crosstalk, promoting immunosuppression and immunotherapy resistance (21). Together, these studies support a conceptual shift from viewing metabolism as a background feature of tumor growth to recognizing tumor-derived metabolites as active immune checkpoints that shape the response to cancer immunotherapy.

To strengthen the conceptual novelty of this mini review, we propose a multilayer model of metabolic immune checkpoints. In this framework, tumor-derived metabolites suppress antitumor immunity through five interconnected mechanisms: metabolite accumulation, enzymatic metabolite conversion, metabolite-sensing receptor signaling, metabolic-epigenetic imprinting, and myeloid education. Lactate, adenosine, tryptophan-derived metabolites, and UDP-related nucleotide signals are therefore not discussed as isolated metabolic products, but as representative nodes within a metabolite-centered immune checkpoint network. This model helps explain how metabolic stress is converted into T-cell dysfunction, Treg enrichment, NK-cell suppression, myeloid reprogramming, spatial immune exclusion, and resistance to immune checkpoint blockade.

Although this mini review focuses on lactate, adenosine, tryptophan-derived metabolites, and nucleotide-derived UDP signaling as representative metabolite-centered immune checkpoints, these pathways do not operate in isolation. Other metabolic programs, including lipid metabolites, reactive oxygen species, arginine metabolism, glutamine metabolism, and mitochondrial redox stress, also contribute to immune suppression and resistance to immune checkpoint blockade. Rather than attempting to exhaustively catalogue all immunometabolic pathways, we use these selected examples to propose an integrated model in which tumor-derived metabolites suppress antitumor immunity through metabolite accumulation, enzymatic conversion, receptor sensing, epigenetic imprinting, and myeloid education. Within this model, lipid peroxidation and ROS can impair antigen presentation and T-cell fitness, arginine depletion can restrict T-cell proliferation and cytotoxicity, and glutamine rewiring can support tumor growth, macrophage polarization, and metabolic adaptation. These additional pathways are therefore discussed as interconnected components of a broader metabolic checkpoint network.

## 2 Lactate as an accumulation–sensing–imprinting checkpoint

Among tumor-derived metabolites, lactate represents one of the most extensively studied and conceptually important metabolic immune checkpoints. For a long time, lactate was mainly regarded as the final product of aerobic glycolysis, reflecting the Warburg phenotype of rapidly proliferating tumor cells. However, this view is now too limited. Accumulating evidence indicates that lactate is not merely a metabolic waste product, but an active signaling molecule that reshapes immune cell recruitment, differentiation, effector function, and therapeutic response within the tumor microenvironment. In highly glycolytic tumors, excessive lactate production is usually accompanied by extracellular acidification, nutrient competition, hypoxia adaptation, and stromal remodeling. These changes collectively create a hostile microenvironment for cytotoxic T cells and natural killer cells, while favoring the accumulation of regulatory T cells, suppressive myeloid cells, and immune-tolerant stromal compartments. Therefore, lactate can be viewed as a central metabolic checkpoint that links tumor-intrinsic glycolysis with immune escape and immunotherapy resistance.

### 2.1 Lactate accumulation links glycolysis to Treg-mediated immune resistance

One major mechanism by which lactate suppresses antitumor immunity is the promotion of regulatory T cell recruitment and persistence. Tregs are metabolically adaptable immune cells that can survive in nutrient-depleted and lactate-rich environments more efficiently than many effector T cells. In contrast, CD8^+^ T cells require coordinated glycolytic and mitochondrial programs to sustain proliferation, cytokine production, and cytotoxic activity (29–31). When tumor cells consume glucose and export lactate, the resulting microenvironment may simultaneously deprive effector T cells of metabolic substrates and provide signals that favor Treg accumulation. This creates a metabolic imbalance in which immunosuppressive cells gain a local survival and functional advantage over antitumor effector cells.

A recent study in gastric cancer provides direct experimental support for this concept. Su et al. showed that highly glycolytic gastric cancer cells produce abundant lactate and that lactate functions as a receptor-mediated immunosuppressive signal rather than a passive glycolytic by-product (32). Mechanistically, lactate acted through GPR81, a lactate-sensing G-protein-coupled receptor, to regulate CX3CL1 expression and promote Treg recruitment. This lactate/GPR81/CX3CL1 axis contributed to an immune-resistant phenotype in highly glycolytic gastric cancer (32). The importance of this study lies in its clear connection between tumor glycolysis, a defined lactate-sensing receptor, chemokine remodeling, and Treg-mediated immune suppression. It moves the field beyond the general observation that “lactate is immunosuppressive” and provides a specific molecular pathway through which lactate organizes an immune-resistant tumor niche. Beyond the GPR81–CX3CL1 axis, lactate-dependent immune suppression should also be viewed as a networked metabolic state (32). LDHA-driven lactate overproduction and extracellular acidification can broadly impair cytotoxic T-cell and NK-cell function while favoring Treg persistence and suppressive myeloid programs (33, 34). Importantly, lactate-rich tumors may also amplify other metabolic checkpoints. For example, glycolytic remodeling and lactate accumulation can coexist with CD39/CD73-adenosine signaling, linking glucose metabolism to purinergic immune suppression. In glioma, recent reviews of the metabolic-immune axis have emphasized that glycolysis, amino acid metabolism, lipid metabolism, and nucleotide metabolism jointly reshape immune infiltration, immune evasion, and therapeutic response (35). Thus, lactate should not be considered a single-pathway suppressor, but a hub metabolite that connects acidification, receptor signaling, lactylation, adenosine production, and myeloid remodeling.

This mechanism is particularly relevant to immunotherapy resistance. Even when tumor antigens are present and T cells have infiltrated the tumor, the local enrichment of Tregs can blunt effective cytotoxic immunity by producing suppressive cytokines, consuming interleukin-2, inhibiting antigen-presenting cells, and restraining CD8^+^ T-cell activation. Lactate-driven Treg recruitment therefore acts in parallel with classical checkpoint pathways. While PD-1/PD-L1 signaling directly inhibits effector T-cell activation, lactate reshapes the cellular composition and metabolic tone of the microenvironment in favor of immune tolerance. In this sense, lactate can function as a “field effect” checkpoint: it does not act on one receptor–ligand pair alone, but reprograms the broader immune ecology of the tumor.

### 2.2 Genomic–metabolic coupling can create lactate-rich immune-suppressive niches

Lactate-mediated immune suppression is often attributed to the general Warburg effect, but recent evidence suggests that it can also be driven by specific tumor-intrinsic genetic alterations (36–39). This is important because it links genomic changes to metabolic immune resistance. Certain mutations, deletions, or pathway abnormalities may enhance glycolytic flux, increase lactate production, and generate local immune-suppressive niches. These niches are not simply the consequence of rapid tumor growth; they can be genetically programmed states that determine how the tumor interacts with immune cells and responds to therapy.

A recent study in hepatocellular carcinoma illustrates this principle. ACVR2A attenuation was linked to hyperglycolytic reprogramming, increased lactate production, enhanced Treg attraction, and reduced sensitivity to PD-1 blockade (25). This finding supports the idea that tumor-intrinsic genetic events can generate lactate-dependent immune resistance niches. In this setting, loss or attenuation of a regulatory pathway does not merely alter cancer cell proliferation; it rewires the metabolic output of tumor cells and indirectly reshapes antitumor immunity. The resulting lactate-rich microenvironment favors Treg accumulation and weakens the efficacy of immune checkpoint blockade. This study is useful for the conceptual development of metabolic immune checkpoints because it connects three layers of resistance: tumor genetics, metabolic reprogramming, and immune exclusion or suppression. The ACVR2A example should be interpreted as a model of genomic–metabolic–immune coupling rather than as a fully established clinical biomarker. It suggests that tumor-intrinsic genetic or signaling abnormalities may reshape metabolic output, increase lactate production, recruit suppressive immune populations, and thereby weaken the efficacy of PD-1 blockade (25, 40). However, the prevalence of ACVR2A attenuation varies across tumor types and clinical cohorts, and its predictive value for immunotherapy response has not yet been systematically validated (41). Therefore, ACVR2A status alone should not currently be considered a mature biomarker for selecting metabolic checkpoint therapy. Instead, it may serve as a candidate component of a composite biomarker strategy that integrates tumor genomics, glycolytic activity, lactate-related gene expression, Treg or myeloid infiltration, spatial metabolite distribution, and clinical response to immune checkpoint blockade (42, 43). Future studies using TCGA, cBioPortal, single-cell datasets, spatial profiling, and ICB-treated clinical cohorts are needed to determine whether specific genomic alterations can reliably identify tumors dependent on lactate-mediated immune resistance.

Classical biomarker models of ICB response often focus on tumor mutational burden, PD-L1 expression, interferon signaling, or T-cell infiltration. However, the ACVR2A-related findings suggest that genetic lesions may also influence immunotherapy response by changing metabolite production (44). A tumor with high glycolytic activity and lactate accumulation may remain resistant to PD-1 blockade even if immune recognition is present, because the metabolic context prevents effective T-cell function and favors regulatory immune circuits. More broadly, tumor-intrinsic oncogenic programs can stabilize lactate-rich immune-suppressive niches by sustaining glycolytic flux. Transcriptional regulators such as HIF1α, MYC, and FOXM1 may enhance glycolytic enzyme expression, including LDHA, thereby coupling genetic or signaling aberrations to persistent lactate production and reduced responsiveness to PD-1 blockade.

These observations also have therapeutic implications. If genetic alterations generate lactate-rich immune-suppressive niches, then metabolic intervention may need to be tailored to the underlying tumor state. Potential strategies include inhibiting lactate production, blocking lactate export, targeting lactate-sensing pathways, or combining glycolytic/lactate-directed therapies with PD-1/PD-L1 blockade. Although these approaches require careful clinical validation, they highlight a broader principle: overcoming immunotherapy resistance may require targeting both immune receptors and tumor metabolic outputs.

### 2.3 Lactate-to-lactylation axis expands metabolic checkpoints into protein modification

The immunoregulatory role of lactate is not limited to extracellular signaling. A major recent advance is the recognition that lactate can also influence cellular behavior through lactylation, a post-translational modification that links metabolic state to gene regulation and protein function. This concept expands the definition of metabolic immune checkpoints. Lactate is not only a soluble metabolite that activates receptors or acidifies the extracellular space; it can also serve as a substrate or signal for intracellular protein modification, thereby altering transcriptional programs, immune cell states, and therapeutic resistance (45–48). This lactate-to-lactylation axis is particularly relevant in tumors with intense glycolysis and poor perfusion, such as pancreatic ductal adenocarcinoma. A recent study reported that elevated protein lactylation promotes an immunosuppressive microenvironment and therapeutic resistance in pancreatic ductal adenocarcinoma (26). This finding extends lactate biology from extracellular metabolic signaling to intracellular post-translational regulation. In pancreatic cancer, where dense stroma, hypoxia, poor immune infiltration, and myeloid-rich suppression are common features, lactylation may provide a mechanism by which metabolic stress is translated into durable immune and therapeutic resistance programs. It is important to distinguish extracellular lactate signaling from intracellular lactylation. Extracellular lactate mainly acts as a microenvironmental signal through acidification, lactate transporters such as MCT1/MCT4, and lactate-sensing receptors such as GPR81/HCAR1 (49, 50). These mechanisms rapidly affect immune-cell metabolism, trafficking, and function, including CD8^+^ T-cell exhaustion, NK-cell suppression, Treg persistence, and macrophage polarization (32). By contrast, intracellular lactylation converts lactate abundance into a more durable regulatory program. Lactate-derived lactyl groups can modify histone proteins, such as H3K18la and H3K9la, as well as non-histone proteins, thereby altering chromatin accessibility, transcription-factor recruitment, protein stability, localization, and enzymatic activity (51, 52). Enzymes such as p300 have been implicated as lactylation writers, whereas HDACs and SIRT family members may function as delactylation erasers (53, 54). Therefore, extracellular lactate signaling mainly reshapes immune-cell behavior at the metabolic and receptor-signaling level, whereas intracellular lactylation imprints longer-lasting transcriptional and phenotypic states in tumor cells, stromal cells, and immune cells. In this context, lactylation should be interpreted as a metabolic-epigenetic checkpoint rather than simply a downstream marker of glycolysis. It can regulate immune-related gene programs, including checkpoint ligand expression, inflammatory mediators, chemokine networks, and myeloid-polarizing signals, thereby linking lactate accumulation to persistent immune suppression and therapeutic resistance.

The significance of lactylation lies in its ability to connect metabolic abundance with functional cell-state changes. While extracellular lactate can act through receptors such as GPR81, intracellular lactylation may alter gene expression or protein function in tumor cells, macrophages, stromal cells, or other immune compartments. This creates a more stable form of metabolic memory, in which a lactate-rich environment imprints suppressive or therapy-resistant phenotypes. Such a mechanism may help explain why some tumors remain refractory to therapy even when individual signaling pathways are blocked. The metabolic state itself can be encoded into regulatory programs through post-translational modification.

From the perspective of cancer immunotherapy, lactylation also broadens the target space. Targeting lactate biology may not be limited to inhibiting glycolysis or lactate transport. It may also involve identifying the enzymes, substrates, and downstream transcriptional programs involved in lactylation-dependent immune suppression. This is especially important for tumors such as pancreatic cancer, where conventional ICB has shown limited efficacy and where metabolic-stromal barriers strongly restrict immune activation.

**Figure 1:**
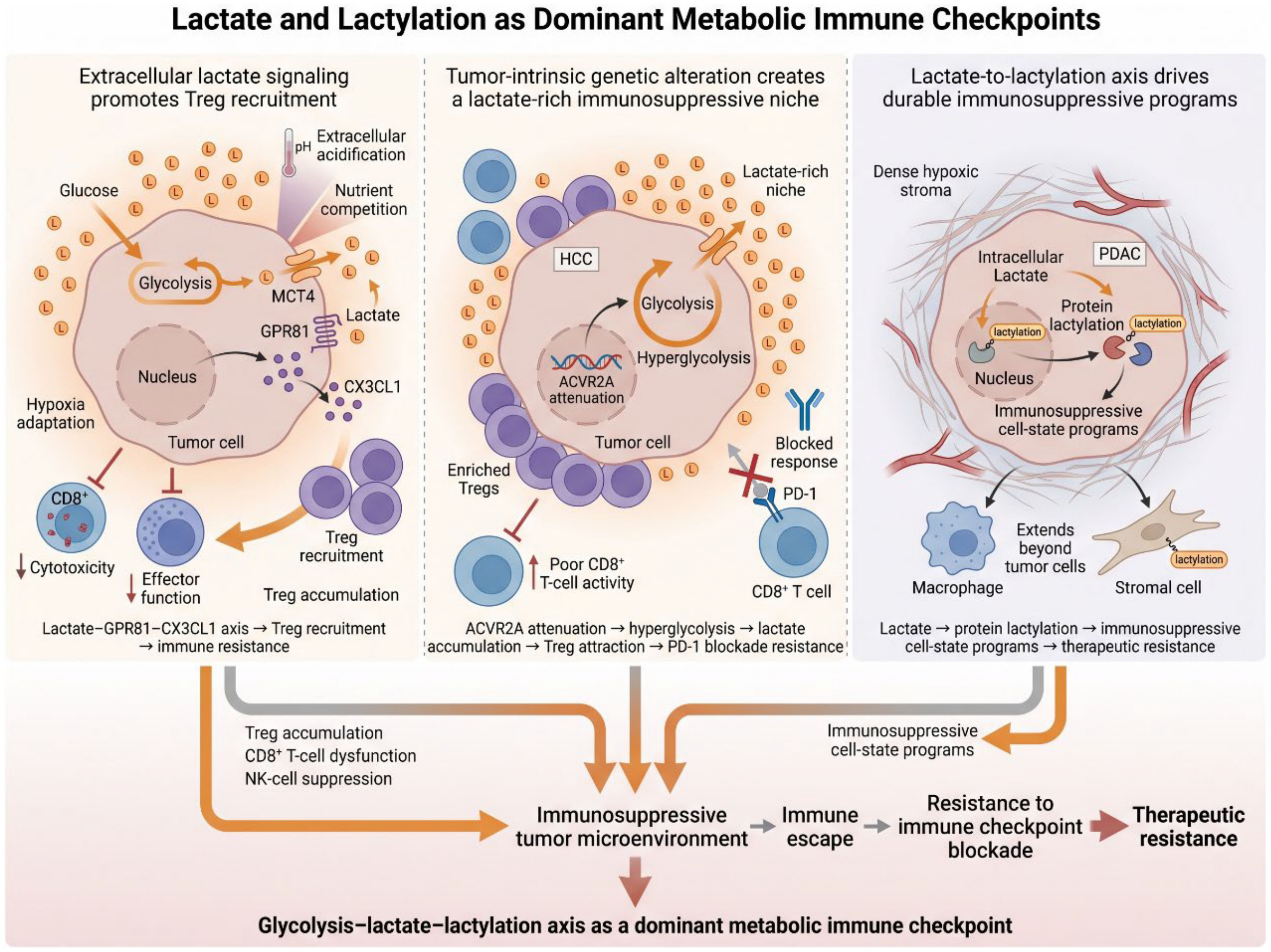
Lactate and lactylation as dominant metabolic immune checkpoints. Highly glycolytic tumor cells produce lactate, which promotes Treg recruitment through the GPR81–CX3CL1 axis, suppresses CD8^+^ T-cell and NK-cell activity, and contributes to immune resistance. Tumor-intrinsic alterations such as ACVR2A attenuation further generate lactate-rich suppressive niches, while lactate-driven protein lactylation imprints durable immunosuppressive programs and therapeutic resistance.

Taken together, these studies establish lactate as a dominant metabolic immune checkpoint operating at multiple levels. First, lactate acts as an extracellular signaling molecule that promotes Treg recruitment through receptor-mediated chemokine remodeling, as shown in gastric cancer. Second, lactate-rich immune-suppressive niches can be generated by tumor-intrinsic genetic alterations, as illustrated by ACVR2A attenuation in hepatocellular carcinoma. Third, lactate can be converted into intracellular regulatory information through protein lactylation, contributing to immunosuppressive microenvironments and therapeutic resistance in pancreatic ductal adenocarcinoma. These mechanisms collectively support a shift from viewing lactate as a passive marker of glycolysis to recognizing it as an active organizer of immune escape. In this framework, the glycolysis–lactate–lactylation axis represents one of the clearest examples of how tumor metabolism can function as an immune checkpoint and shape resistance to cancer immunotherapy. **Figure 1** illustrates how the glycolysis–lactate–lactylation axis connects tumor metabolic reprogramming with immune suppression and resistance to immune checkpoint blockade. Lactate acts both as an extracellular signaling molecule that remodels immune-cell recruitment and as an intracellular driver of lactylation-dependent resistant cell states.

## 3 The CD39/CD73-adenosine axis as an enzymatic conversion checkpoint

Adenosine represents one of the most established enzymatically generated metabolic immune checkpoints in cancer. In contrast to lactate, which mainly reflects glycolytic overflow and acidification, adenosine is produced through a defined extracellular nucleotide catabolic cascade. Under conditions of hypoxia, tissue stress, cell death, and inflammation, extracellular ATP is released into the tumor microenvironment. Although ATP can initially function as a danger signal that promotes immune activation, it is rapidly converted into immunosuppressive adenosine through the sequential activity of ectonucleotidases. CD39 hydrolyzes ATP and ADP to AMP, while CD73 further converts AMP into adenosine (55–58). The resulting adenosine-rich microenvironment suppresses antitumor immunity through adenosine receptors, particularly A2A and A2B receptors, expressed on immune and stromal cells. The immunological consequences of adenosine accumulation are broad. Adenosine can inhibit CD8^+^ T-cell receptor signaling, reduce cytokine production, impair cytotoxic activity, and limit T-cell proliferation. It also suppresses natural killer cell activity, weakens dendritic cell maturation, and promotes the expansion or functional stability of regulatory T cells and suppressive myeloid cells. In this way, the CD39/CD73-adenosine pathway converts inflammatory or damage-associated nucleotide signals into an immunoregulatory program (59–61). This is highly relevant to immune-excluded and myeloid-rich tumors, where hypoxia, necrosis, poor perfusion, and stromal stress continuously generate extracellular nucleotides. Instead of sustaining immune activation, these signals may be enzymatically redirected toward adenosine-mediated immune tolerance.

### 3.1 CD73-mediated adenosine production limits anti-PD-1 response

CD73 is a particularly important node in this pathway because it controls the final step of extracellular adenosine generation. High CD73 expression in tumor cells, stromal cells, endothelial cells, or immune suppressive populations can amplify local adenosine production and thereby weaken the efficacy of immune checkpoint blockade. This makes CD73 not only a marker of immunosuppressive metabolism but also a potential therapeutic target. In the context of anti-PD-1 therapy, CD73-mediated adenosine signaling may create a parallel inhibitory circuit. Even if PD-1 signaling is blocked, adenosine can continue to suppress T-cell activity through A2A/A2B receptor signaling, limiting the full restoration of antitumor immunity.

A recent study in intrahepatic cholangiocarcinoma provides direct experimental support for this concept. In this study, targeting CD73 limited tumor progression and enhanced the antitumor activity of anti-PD-1 therapy (62). This finding is important because it places CD73 within a clinically relevant resistance framework. Rather than simply showing that CD73 is associated with immune suppression, the study demonstrates that interfering with the adenosine-generating machinery can improve the response to PD-1 blockade (63–65). Therefore, CD73 functions as a targetable metabolic immune checkpoint that operates alongside classical immune checkpoints. This evidence is especially relevant for tumors such as intrahepatic cholangiocarcinoma, which often display dense stromal components, inflammatory remodeling, immune exclusion, and limited responsiveness to immunotherapy. In such tumors, resistance may not be explained solely by insufficient antigenicity or checkpoint expression. Instead, the local enzymatic conversion of extracellular nucleotides into adenosine may establish a suppressive immune tone that prevents effective T-cell reinvigoration. Targeting CD73 may therefore help convert an adenosine-dominated suppressive niche into a more permissive environment for anti-PD-1-mediated immune activation.

The therapeutic implication is straightforward but important: immune checkpoint blockade may require simultaneous disruption of metabolic checkpoints. Anti-PD-1 therapy primarily releases inhibitory signaling on exhausted or dysfunctional T cells, whereas CD73 blockade reduces the accumulation of adenosine that suppresses immune cells through metabolic receptor signaling. The combination therefore addresses two distinct but cooperating layers of immune suppression. This rationale supports the broader concept that metabolic enzymes within the tumor microenvironment can act as functional checkpoints and should be considered in combination immunotherapy design.

### 3.2 NETs and myeloid inflammation amplify the CD73-adenosine checkpoint

Although CD73 is often discussed as a tumor-intrinsic or stromal metabolic enzyme, its regulation is strongly influenced by inflammatory and myeloid processes. This is important because many tumors are not metabolically suppressive only because of cancer cell-intrinsic programs. They also contain inflammatory immune structures that reinforce suppressive metabolism. Among these structures, neutrophil extracellular traps (NETs) have emerged as important contributors to tumor progression, metastasis, inflammation, and immune escape (66–69). NETs are web-like extracellular structures composed of DNA, histones, and granular proteins released by activated neutrophils. In tumors, NETs can remodel the extracellular matrix, trap circulating tumor cells, promote thrombosis-like inflammatory states, and interact with tumor and immune cells.

A recent hepatocellular carcinoma study further connected NETs to the adenosine checkpoint by showing that neutrophil extracellular traps promote immune escape through Notch2-mediated upregulation of CD73 (27). This finding adds an important layer to the CD73-adenosine framework. It suggests that adenosine-mediated immune suppression can be amplified by neutrophil-driven inflammatory programs, rather than being solely determined by baseline CD73 expression in tumor cells. Through Notch2-dependent CD73 upregulation, NETs may increase the capacity of the tumor microenvironment to generate adenosine and suppress antitumor immunity. This mechanism is particularly meaningful in hepatocellular carcinoma, a tumor type frequently characterized by chronic inflammation, liver injury, immune tolerance, and abundant myeloid cell infiltration. In such a setting, neutrophils and NETs may serve as a bridge between inflammation and metabolic immune escape. In principle, inflammation should promote immune activation. However, when inflammatory neutrophil programs induce CD73, the result may shift from antitumor immunity toward adenosine-mediated tolerance. This illustrates a paradox of the tumor microenvironment: inflammatory signals can be metabolically reprogrammed into immunosuppressive outputs.

The NETs-Notch2-CD73 axis also broadens the therapeutic perspective. Targeting CD73 alone may be useful, but upstream inflammatory drivers such as NET formation, neutrophil activation, or Notch2 signaling may also influence the strength of adenosine-mediated suppression. Therefore, combined strategies that interrupt both myeloid inflammation and adenosine production may be particularly relevant for immune-excluded or myeloid-dominant tumors. Such approaches could include anti-CD73 therapy, A2A/A2B receptor blockade, NET-disrupting strategies, or combinations with anti-PD-1/PD-L1 therapy. The upstream signals that activate the NETs–Notch2–CD73 circuit are likely to be context-dependent. In hepatocellular carcinoma, chronic liver injury, viral or metabolic inflammation, hypoxia, necrosis, neutrophil recruitment, and damage-associated inflammatory signals may jointly promote NET formation and strengthen tumor–neutrophil interactions. NET-derived DNA, histones, and granular proteins can then provide a local inflammatory scaffold that activates Notch2-related signaling in tumor cells and enhances CD73 expression. This mechanism may be particularly relevant to HCC because the liver is characterized by chronic inflammatory injury, tolerogenic immune regulation, sinusoidal myeloid-cell enrichment, and frequent hypoxic or necrotic tumor regions. Therefore, the NETs–Notch2–CD73 axis should not be assumed to operate uniformly across all cancers. Its activity may depend on tissue-specific inflammatory tone, neutrophil abundance, stromal architecture, and the baseline expression of Notch receptors and CD73.

**Figure 2:**
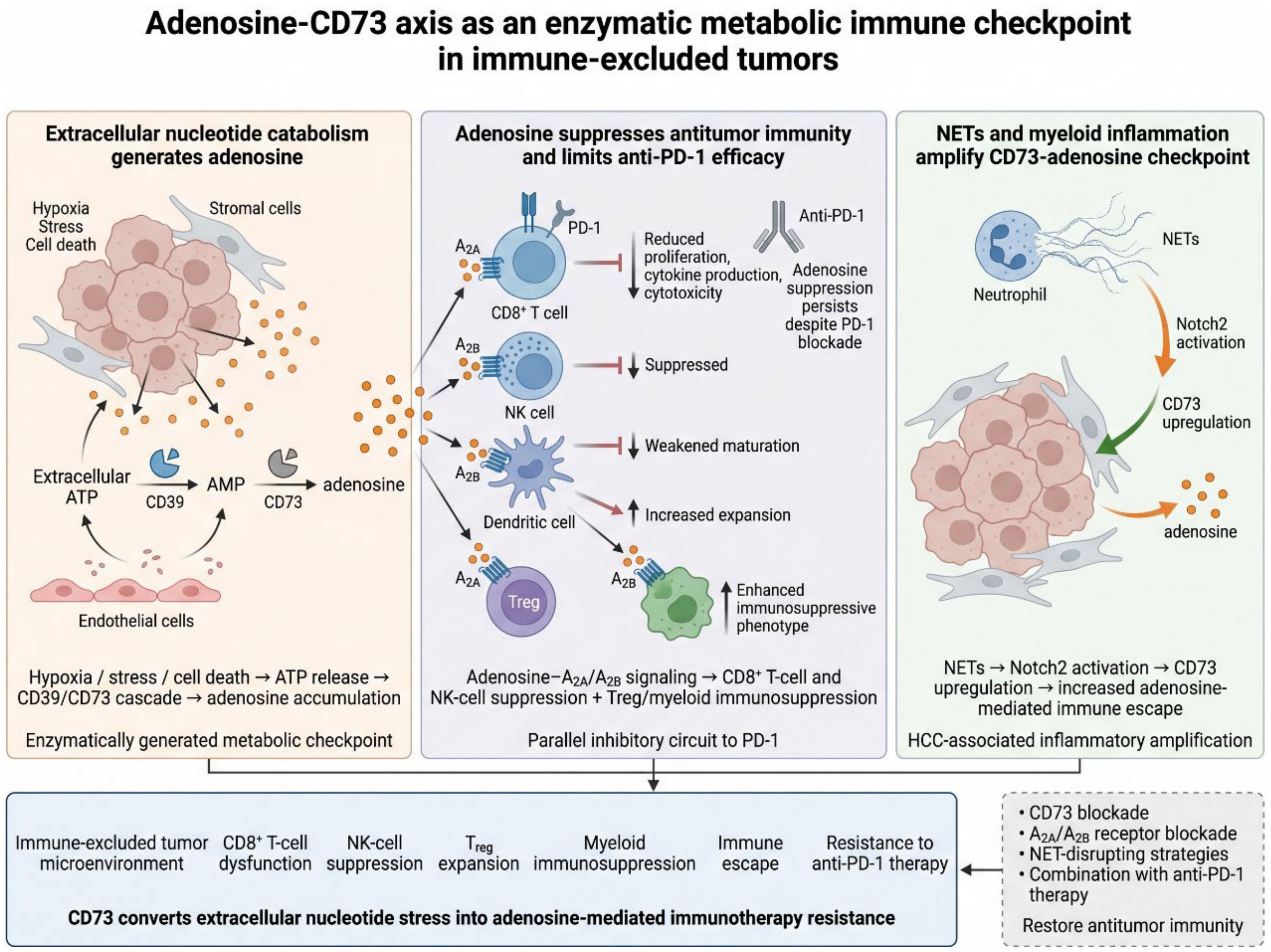
Adenosine-CD73 axis as an enzymatic metabolic immune checkpoint. Hypoxia, inflammation, necrosis, and tissue stress promote extracellular ATP release, which is sequentially converted by CD39 and CD73 into immunosuppressive adenosine. Adenosine signals through A2A/A2B receptors to suppress CD8^+^ T cells, NK cells, and dendritic cells while promoting Tregs and suppressive myeloid cells. NETs further amplify CD73 expression through Notch2, reinforcing immune escape and anti-PD-1 resistance.

Taken together, these studies position the CD73-adenosine axis as a prototypical enzymatic metabolic immune checkpoint. It differs from lactate-centered suppression in that adenosine is generated through a defined extracellular enzyme cascade and acts through specific purinergic receptors. However, both pathways converge on a similar outcome: suppression of cytotoxic immunity and reinforcement of regulatory or myeloid-dominant immune states. The intrahepatic cholangiocarcinoma study demonstrates that CD73 targeting can limit tumor progression and enhance anti-PD-1 efficacy, while the hepatocellular carcinoma NET study shows that inflammatory neutrophil structures can amplify CD73-dependent immune escape. These findings support a model in which enzymatic metabolite production, stromal-inflammation interactions, and classical checkpoint resistance are tightly interconnected. In this framework, CD73 is not merely a metabolic enzyme but a functional immune checkpoint that converts extracellular nucleotide stress into adenosine-mediated immunotherapy resistance. **Figure 2** illustrates how extracellular nucleotide stress is enzymatically redirected into adenosine-mediated immune suppression through the CD39/CD73 pathway. It also highlights how NET-driven Notch2 activation amplifies CD73 expression, linking myeloid inflammation with immune exclusion and resistance to anti-PD-1 therapy.

## 4 Tryptophan metabolism as a networked metabolite-sensing checkpoint

Tryptophan metabolism is one of the earliest and most intensively studied metabolic pathways involved in tumor immune escape (70, 71). In the classical model, tumor cells, antigen-presenting cells, or stromal cells upregulate indoleamine 2, 3-dioxygenase 1 (IDO1), IDO2, or tryptophan 2, 3-dioxygenase (TDO), leading to local tryptophan depletion and accumulation of kynurenine and other downstream metabolites. These changes can suppress antitumor immunity through two interconnected mechanisms. First, tryptophan depletion activates nutrient stress responses in effector T cells, limiting proliferation and cytokine production. Second, kynurenine and related metabolites activate immunoregulatory signaling pathways, particularly aryl hydrocarbon receptor (AhR), which can promote regulatory T cell differentiation, impair cytotoxic T-cell function, and support tolerogenic myeloid states. Therefore, the tryptophan–kynurenine pathway has long been viewed as a prototypical metabolic immune checkpoint. However, clinical attempts to target this pathway have shown that tryptophan metabolism is more complex than a single IDO1-centered axis. The limited success of some IDO1 inhibitor combinations suggests that immune escape may not depend only on one enzyme or one downstream metabolite. Instead, tumors may use multiple tryptophan-metabolic branches, alternative enzymes, compensatory pathways, and non-classical immune checkpoints to maintain immune suppression. Recent studies have therefore shifted the field from a narrow focus on IDO1 inhibition toward a broader view of tryptophan-derived metabolic networks. In this updated framework, kynurenine, indole derivatives, serotonin-related metabolites, and their downstream immune sensors may cooperate to shape tumor immunity and resistance to immune checkpoint blockade.

### 4.1 Kynurenine connects tumor metabolism with Siglec-15-mediated immune escape

A particularly important development is the connection between kynurenine metabolism and non-classical immune checkpoint molecules. Traditionally, kynurenine has been discussed mainly in relation to AhR activation. Although this remains highly relevant, emerging evidence indicates that kynurenine may also interact with additional immune regulatory pathways (72, 73). This expands the role of tryptophan-derived metabolites from general immune suppression to more specific checkpoint-like signaling circuits.

In head and neck squamous cell carcinoma, metabolic profiling identified a novel kynurenine/Siglec-15 axis that contributes to immune escape (74). This study is especially useful for the present review because it links tumor metabolic state with a non-PD-1 immune checkpoint pathway. Siglec-15 has attracted attention as an immunosuppressive molecule that may operate in tumors with low PD-L1 expression or resistance to PD-1/PD-L1 blockade. By connecting kynurenine metabolism to Siglec-15-associated immune escape, this work suggests that tryptophan-derived metabolites may cooperate with alternative checkpoint pathways beyond the canonical IDO1–AhR model.

The conceptual significance of this finding is substantial. In many tumors, resistance to PD-1 blockade cannot be explained solely by the absence of T cells or lack of PD-L1 expression. Instead, alternative suppressive circuits may dominate. A kynurenine/Siglec-15 axis provides one possible mechanism by which tumor metabolism can reinforce non-classical immune checkpoint activity. In this setting, kynurenine is not only a soluble immunosuppressive metabolite but also part of a broader checkpoint ecosystem. It may help establish a tumor microenvironment in which T-cell activation remains limited even when PD-1/PD-L1 signaling is therapeutically blocked (75, 76). This mechanism also highlights the value of metabolic profiling for discovering immune escape pathways. Rather than starting from a known immune receptor, the study used the metabolic landscape of head and neck squamous cell carcinoma to identify an immune regulatory axis. This approach is aligned with the concept of metabolic immune checkpoints: tumor-derived metabolites can reveal hidden immune suppressive programs that are not obvious from classical immune-marker analysis alone. For mini review framing, this study can be used to argue that future biomarker discovery should integrate metabolomics with immune checkpoint profiling, spatial immune analysis, and functional validation. Therapeutically, the kynurenine/Siglec-15 axis suggests several possibilities. Tumors with high kynurenine metabolism and Siglec-15-associated immune suppression may require strategies beyond PD-1/PD-L1 blockade. Potential approaches include targeting upstream tryptophan-metabolic enzymes, interfering with kynurenine signaling, blocking Siglec-15, or combining metabolic intervention with alternative checkpoint blockade. Although these strategies still require more clinical validation, the key message is that tryptophan metabolism may define immune escape states that are not fully reversible by classical ICB alone.

However, the kynurenine/Siglec-15 axis should currently be interpreted as an emerging and context-dependent mechanism rather than a universally established tryptophan checkpoint. Most available evidence comes from head and neck squamous cell carcinoma, and whether this axis operates similarly in other tumor types, treatment settings, or immune microenvironmental states remains unclear (74). In addition, the clinical experience with IDO1 inhibition has been inconsistent. For example, the phase III ECHO-301/KEYNOTE-252 trial showed that epacadostat plus pembrolizumab did not improve progression-free survival or overall survival compared with pembrolizumab alone in advanced melanoma (77). These findings indicate that tryptophan metabolism cannot be targeted effectively by assuming a linear IDO1–kynurenine–T-cell suppression model. Future studies should clarify whether Siglec-15-associated kynurenine signaling identifies a distinct subgroup of tumors and whether this mechanism is reproducible across independent cohorts and functional models.

### 4.2 Reprogramming tryptophan metabolism can restore ICB response

Another important advance is the recognition that tryptophan metabolism can be pharmacologically reprogrammed to restore sensitivity to immunotherapy. Earlier strategies focused heavily on direct IDO1 inhibition. However, the tryptophan metabolic network contains several interconnected branches, including kynurenine, indole, and serotonin pathways. These branches may have distinct but overlapping effects on tumor cells, immune cells, microbial interactions, and systemic inflammatory tone. Therefore, effective therapeutic intervention may require broader remodeling of tryptophan metabolism rather than inhibition of a single enzymatic node.

A recent ovarian cancer study supports this view. Amitriptyline was reported to revitalize ICB response by simultaneously inhibiting kynurenine/indole and serotonin branches of tryptophan metabolism (28). This finding is important for two reasons. First, it provides experimental evidence that tryptophan-derived metabolic programs can actively limit ICB efficacy. Second, it suggests that a repurposed pharmacological agent can reshape multiple tryptophan-metabolic branches and improve response to immune checkpoint blockade. This moves the field beyond the simplistic model of “IDO1 inhibition plus anti-PD-1” and toward a more network-based approach to metabolic immunotherapy.

The ovarian cancer context is also meaningful. Ovarian cancer is often characterized by an immunosuppressive tumor microenvironment, frequent T-cell dysfunction, suppressive myeloid and stromal compartments, and relatively modest responses to ICB in many clinical settings (78–81). If tryptophan-derived metabolites contribute to this suppressive state, then metabolic reprogramming may help convert an immune-resistant tumor into a more immunotherapy-responsive state. By targeting both kynurenine/indole and serotonin branches, amitriptyline may reduce multiple layers of metabolic immune suppression simultaneously. This study also raises a broader translational point. Metabolic immune checkpoints may be more effectively targeted through systems-level modulation than through single-target blockade. Tumors can compensate for inhibition of one pathway by increasing alternative metabolic branches or using parallel immunosuppressive circuits. Therefore, drug repurposing or combination strategies that influence several related metabolic outputs may be valuable, provided that specificity, toxicity, and immune consequences are carefully evaluated. In this regard, the amitriptyline study provides a useful example of how an existing drug may be repositioned to modulate tumor metabolism and improve ICB efficacy.

Together, these findings indicate that tryptophan-derived metabolites remain central to metabolic immune checkpoint biology, but the field is moving beyond the traditional IDO1-centered model. The kynurenine/Siglec-15 axis in head and neck squamous cell carcinoma demonstrates that tryptophan metabolites can cooperate with non-classical immune checkpoints and may explain resistance states not captured by PD-1/PD-L1 analysis alone. The ovarian cancer study further shows that broader reprogramming of kynurenine/indole and serotonin branches can restore ICB response. Thus, tryptophan metabolism should be viewed not as a single linear pathway, but as a flexible immunoregulatory network. Understanding this network may reveal new biomarkers, alternative checkpoint targets, and rational combinations for overcoming immunotherapy resistance.

## 5 UDP-driven macrophage crosstalk as an emerging myeloid-education checkpoint

Although lactate, adenosine, and tryptophan-derived metabolites represent the best-known metabolic immune checkpoints, the landscape of metabolite-mediated immune regulation is expanding rapidly. A particularly emerging area is nucleotide metabolism (82). Traditionally, nucleotide metabolism has been viewed mainly as a biosynthetic program that supports DNA replication, RNA synthesis, tumor proliferation, and repair after genotoxic stress. Rapidly dividing cancer cells require increased nucleotide production to maintain growth, adapt to oncogenic stress, and survive therapeutic pressure. However, recent evidence suggests that nucleotide metabolism does not only serve tumor-intrinsic biosynthetic needs (83–85). It can also generate extracellular or paracrine metabolic signals that reshape immune cell behavior, especially within myeloid-rich tumor microenvironments. This concept is important because many immunotherapy-resistant tumors are dominated not only by dysfunctional T cells, but also by suppressive macrophages, monocytes, and other myeloid populations. Tumor-associated macrophages (TAMs) can inhibit cytotoxic T-cell activity, reduce antigen presentation, promote angiogenesis, remodel extracellular matrix, and support tumor invasion. In many tumor types, high TAM infiltration is associated with poor response to immune checkpoint blockade. Therefore, identifying metabolic signals that sustain TAM-mediated immune suppression is essential for understanding why some tumors remain resistant to PD-1/PD-L1 blockade even when T cells are present.

A recent Nature Cancer study by Scolaro et al. expanded the landscape of metabolic immune checkpoints by showing that cancer cell nucleotide metabolism fuels UDP-driven crosstalk with macrophages, promoting immunosuppression and immunotherapy resistance (21). This study is highly relevant to the present review because it introduces nucleotide-derived metabolites as active regulators of tumor–immune communication. Rather than treating nucleotide metabolism as a background growth-supporting pathway, the study demonstrates that cancer cells can use nucleotide metabolic outputs to communicate with macrophages and shape an immunosuppressive microenvironment.

The key conceptual advance is that UDP-related signals can function as mediators of tumor–macrophage crosstalk. In this model, enhanced nucleotide metabolism in cancer cells leads to the release or accumulation of UDP-associated extracellular signals. These signals are sensed by macrophages and promote an immunosuppressive state. As a result, macrophages become active participants in therapy resistance rather than passive bystanders. They help establish a tumor microenvironment that limits effective antitumor immunity and reduces the efficacy of immune checkpoint blockade. This places nucleotide-derived metabolites within the broader category of metabolic immune checkpoints. This mechanism differs from the more familiar lactate and adenosine pathways in several ways. Lactate mainly reflects glycolytic overflow and can act through acidification, receptor-mediated signaling, and lactylation. Adenosine is generated through extracellular nucleotide catabolism by CD39 and CD73 and suppresses immune cells through adenosine receptors. UDP-driven macrophage crosstalk, by contrast, highlights a more specific form of metabolite-mediated intercellular communication between cancer cells and myeloid cells. It suggests that tumor metabolism can create immune resistance not only by globally suppressing T cells, but also by instructing macrophages to adopt suppressive programs. This is especially important for myeloid-dominant resistant niches. In many solid tumors, TAMs are spatially enriched in hypoxic, necrotic, stromal, or perivascular regions where they interact closely with cancer cells and extracellular metabolic signals. These macrophage-rich areas can exclude cytotoxic lymphocytes, suppress local antigen presentation, and produce cytokines or growth factors that sustain tumor progression. UDP-driven communication may therefore represent one mechanism by which tumor cells metabolically educate macrophages within these resistant niches. From this perspective, nucleotide metabolism becomes part of the immune architecture of the tumor microenvironment, not just a cancer cell-autonomous growth pathway.

The therapeutic implications are significant. If nucleotide-derived metabolites support macrophage-mediated immunosuppression, then targeting nucleotide metabolism or UDP-sensing pathways may improve immunotherapy response. Potential strategies could include inhibiting nucleotide biosynthesis, blocking extracellular UDP-related signaling, interfering with macrophage sensing of nucleotide metabolites, or combining such approaches with macrophage reprogramming and PD-1/PD-L1 blockade. Importantly, these strategies would need to be carefully designed because nucleotide metabolism is also essential for normal proliferating cells and immune activation. Therefore, the goal should not be indiscriminate suppression of nucleotide metabolism, but selective disruption of tumor-driven metabolite signals that sustain immunosuppressive macrophage states.

The study by Scolaro et al. also provides a useful conceptual bridge between metabolism and myeloid checkpoints. In classical immunotherapy, checkpoint resistance is often viewed through the lens of T-cell exhaustion. However, in many tumors, the dominant barrier may be a suppressive myeloid ecosystem that prevents T cells from functioning properly. UDP-driven macrophage crosstalk supports the idea that tumor-derived metabolites can operate upstream of T-cell dysfunction by shaping the myeloid compartment. This broadens the definition of metabolic immune checkpoints from direct T-cell inhibitory signals to metabolite-mediated cellular circuits that organize immune-suppressive niches.

Overall, nucleotide metabolism and UDP-driven macrophage crosstalk represent an emerging and highly novel branch of metabolic immune checkpoint biology. This direction is valuable for the present mini review because it moves beyond the well-established lactate, adenosine, and tryptophan pathways and highlights how tumor-derived metabolites can regulate macrophage-dominant immune resistance. By positioning nucleotide-derived metabolites as organizers of myeloid suppressive niches, this section emphasizes that metabolic immune checkpoints are not limited to classic small-molecule suppressors. They also include metabolite-driven intercellular communication programs that connect cancer cell biosynthesis, macrophage polarization, and resistance to immune checkpoint blockade.

Despite its conceptual importance, UDP-driven macrophage crosstalk remains at an early stage of validation. Compared with lactate, adenosine, and tryptophan metabolism, which have been investigated across multiple tumor types and experimental systems, the UDP–macrophage axis is supported by more limited evidence and requires independent confirmation. Several issues remain unresolved, including whether extracellular UDP accumulation is a common feature of immunotherapy-resistant tumors, whether P2Y6-dependent macrophage programming is conserved across cancer types, and whether targeting this pathway can improve immune checkpoint blockade without impairing physiological nucleotide metabolism or normal immune-cell function. Therefore, UDP-driven signaling should be presented as an emerging myeloid metabolic checkpoint rather than a broadly established resistance mechanism.

## 6 Therapeutic implications: combining metabolic checkpoint blockade with immunotherapy

The recognition of tumor-derived metabolites as metabolic immune checkpoints has important therapeutic implications. Current immunotherapy combinations are still largely centered on immune receptors, cytokines, costimulatory molecules, or tumor antigen presentation. However, the studies discussed above suggest that immune resistance is also maintained by metabolite-producing tumor programs and metabolite-sensing immune circuits. Therefore, the next generation of immunotherapy combinations may need to target not only inhibitory receptors on T cells but also the metabolic conditions that prevent immune cells from functioning effectively. In this framework, metabolic checkpoint blockade does not replace immune checkpoint blockade, but may provide a complementary strategy to convert metabolically suppressive tumors into immunologically responsive tumors.

A key implication of this framework is that metabolic checkpoints are interconnected rather than independent. Lactate accumulation can reinforce extracellular acidification, lactylation-dependent transcriptional imprinting, and adenosine-associated immune suppression. Adenosine signaling can cooperate with hypoxia and myeloid inflammation to maintain immune exclusion. Tryptophan-derived kynurenine and indole metabolites may converge with lactate-driven epigenetic remodeling and AhR-dependent immune regulation to stabilize Treg- and TAM-enriched niches. In parallel, arginine depletion, glutamine rewiring, lipid metabolites, and ROS-dependent oxidative stress may further restrict T-cell fitness, impair antigen presentation, and support suppressive myeloid states. Therefore, therapeutic strategies should not target a single metabolite in isolation, but should identify the dominant metabolic checkpoint network in each tumor context.

### 6.1 Therapeutic hierarchy and rational combination logic

Metabolic checkpoint-targeted combinations should be prioritized according to evidence strength, clinical maturity, biomarker availability, and safety feasibility rather than simply listed by pathway. At present, the CD73-adenosine axis has the highest clinical maturity because CD73-directed agents have entered early-phase clinical trials and have been combined with PD-1/PD-L1 blockade or chemotherapy (86–88). However, the clinical benefit appears context-dependent and may require CD73-high, hypoxic, or adenosine-enriched tumors rather than unselected patient populations. Tryptophan-targeted therapy has strong mechanistic rationale but lower current confidence because the failure of epacadostat plus pembrolizumab in phase III melanoma indicates that IDO1 inhibition alone is insufficient without confirming pathway dependence (77). Lactate-targeted therapy, including LDHA, MCT1/MCT4, GPR81/HCAR1, and lactylation-directed strategies, has broad mechanistic relevance but remains largely preclinical in the ICB setting (89, 90). UDP-driven macrophage crosstalk represents the most exploratory category and may be most appropriate for TAM-dominant, myeloid-excluded tumors after further validation (21). Based on this hierarchy, rational combinations can be grouped into four levels. First, clinically advanced combinations include CD73 or adenosine-receptor blockade with anti-PD-1/PD-L1 therapy, particularly in CD73-high or hypoxic tumors. Second, biomarker-dependent combinations include IDO1/TDO/AhR or Siglec-15-associated targeting with ICB in kynurenine-high or AhR-active tumors. Third, metabolism-resetting combinations include LDHA or MCT blockade with ICB to reduce lactate accumulation, extracellular acidification, and lactylation-associated immune resistance. Fourth, myeloid-reprogramming combinations include nucleotide-metabolite targeting with macrophage-directed therapy and ICB in TAM-rich tumors. Therefore, the therapeutic value of each metabolic checkpoint depends not only on its biological importance, but also on whether a dominant immune-metabolic dependency can be identified in a specific tumor context.

### 6.2 Targeting lactate signaling and lactylation

The glycolysis–lactate axis is one of the most attractive therapeutic targets because it operates at several levels of immune suppression. Tumor-derived lactate can promote Treg recruitment, impair effector T-cell activity, reshape myeloid function, acidify the extracellular space, and induce lactylation-dependent regulatory programs. The lactate/GPR81/CX3CL1 axis in gastric cancer demonstrates that lactate can act through a defined receptor-mediated pathway to recruit Tregs and promote immune resistance (32). Similarly, ACVR2A attenuation in hepatocellular carcinoma links tumor-intrinsic genetic alteration with hyperglycolysis, lactate production, Treg attraction, and reduced response to PD-1 blockade (25). In pancreatic ductal adenocarcinoma, elevated protein lactylation further connects lactate abundance with immunosuppressive microenvironment formation and therapeutic resistance (26). These findings suggest several therapeutic entry points. Inhibiting LDHA may reduce lactate production, while targeting MCT1 or MCT4 may limit lactate transport and extracellular accumulation. Blocking GPR81 could disrupt lactate-sensing pathways that promote Treg recruitment or immune suppression. In addition, targeting lactylation-related enzymes, substrates, or downstream transcriptional programs may help reverse more stable lactate-imprinted resistant states. A rational combination strategy would therefore involve pairing anti-PD-1/PD-L1 therapy with interventions that reduce lactate production, lactate export, lactate sensing, or lactylation-mediated immune suppression. However, because lactate metabolism is also involved in normal tissue function and immune cell adaptation, such approaches will require careful selection of tumor types, metabolic biomarkers, and therapeutic windows.

Among lactate-directed strategies, LDHA inhibition has received particular attention because it directly reduces lactate generation. Oxamate, a classical competitive LDHA inhibitor, has shown preclinical activity by disrupting glycolytic metabolism and lactate production (34). In glioblastoma models, oxamate was reported to inhibit stemness-associated phenotypes, including EMT and DNA repair capacity, and to increase radiosensitivity (91). In lung adenocarcinoma, LDHA knockdown or oxamate treatment reduced lactate production and ATP levels and enhanced sensitivity to lobaplatin, supporting the therapeutic relevance of targeting LDHA-dependent metabolic adaptation (92). These findings suggest that LDHA blockade may not only weaken lactate-mediated immune suppression but also improve responses to conventional cytotoxic therapy (93). However, the clinical translation of oxamate-like agents remains limited by metabolic plasticity, potential LDHB compensation, alternative glutamine utilization, and pharmacological constraints such as suboptimal membrane permeability. Future strategies may require optimized delivery systems and rational combinations that simultaneously target lactate production, adenosine signaling, and immune checkpoint pathways.

### 6.3 Targeting the CD73-adenosine axis

The CD39/CD73-adenosine pathway represents a more enzymatically defined metabolic checkpoint. In this pathway, extracellular ATP is converted into immunosuppressive adenosine, which acts through A2A and A2B receptors to inhibit CD8^+^ T cells and NK cells while supporting Tregs and suppressive myeloid compartments. The intrahepatic cholangiocarcinoma study showing that CD73 targeting limits tumor progression and enhances anti-PD-1 activity provides direct evidence that blocking adenosine production can improve ICB efficacy (62). In hepatocellular carcinoma, NETs were shown to upregulate CD73 through Notch2, thereby connecting neutrophil-driven inflammation with adenosine-mediated immune escape (27). Therapeutically, this pathway can be targeted at several levels, including CD39, CD73, A2A receptor, and A2B receptor. Among these, CD73 is particularly attractive because it controls the final extracellular step of adenosine production. Anti-CD73 therapy combined with anti-PD-1 may be especially relevant in immune-excluded or myeloid-rich tumors where adenosine suppresses T-cell reinvigoration. In tumors with strong neutrophil or NET signatures, upstream targeting of NET formation, Notch2 signaling, or inflammatory myeloid activation may further reduce CD73-driven adenosine accumulation. Thus, adenosine blockade may need to be integrated with both ICB and myeloid-directed strategies.

### 6.4 Targeting tryptophan metabolic networks

Tryptophan metabolism has long been considered a therapeutic target, but recent findings suggest that simple IDO1 inhibition may be insufficient. Tumors can use multiple tryptophan-derived branches, including kynurenine, indole, and serotonin pathways, to maintain immune suppression. The kynurenine/Siglec-15 axis identified in head and neck squamous cell carcinoma links tryptophan metabolism with a non-classical immune checkpoint pathway (74). This suggests that tryptophan-derived metabolites may contribute to immune escape states that are not fully addressed by PD-1/PD-L1 blockade alone. In ovarian cancer, amitriptyline was reported to restore ICB response by simultaneously inhibiting kynurenine/indole and serotonin branches of tryptophan metabolism, highlighting the value of broader metabolic reprogramming rather than single-node inhibition (28). These studies support a network-based therapeutic model. Potential strategies include targeting IDO1, TDO, AhR, Siglec-15, or multiple downstream tryptophan-metabolic branches. Drug repurposing may also be useful when existing agents can reshape several metabolic outputs simultaneously. For ICB-resistant tumors, combining PD-1/PD-L1 blockade with inhibitors of kynurenine signaling, Siglec-15 blockade, or broader tryptophan-metabolic reprogramming may help overcome metabolite-driven immune escape. Future studies should define which tumors are dominated by IDO1-dependent, Siglec-15-associated, AhR-mediated, or serotonin-linked suppressive programs.

### 6.5 Targeting nucleotide-metabolite myeloid crosstalk

Nucleotide metabolism provides a newer therapeutic direction. The Nature Cancer study by Scolaro et al. showed that cancer cell nucleotide metabolism fuels UDP-driven crosstalk with macrophages, promoting immunosuppression and immunotherapy resistance (21). This finding suggests that tumor-derived nucleotide metabolites can organize myeloid-dominant resistant niches and suppress ICB efficacy indirectly through TAM programming.

Potential therapeutic strategies include blocking nucleotide synthesis, interfering with extracellular UDP-related signaling, disrupting macrophage sensing of nucleotide metabolites, or combining nucleotide-metabolic intervention with macrophage reprogramming and ICB. Because nucleotide metabolism is essential for normal proliferating cells and immune activation, selective targeting will be critical. Rather than broadly suppressing nucleotide metabolism, future approaches should identify tumor-specific metabolic dependencies or extracellular signaling nodes that sustain immunosuppressive TAM states.

Overall, metabolic checkpoint blockade offers a complementary strategy for overcoming immunotherapy resistance. Lactate-directed therapies may reduce Treg recruitment and lactylation-driven resistance; CD73-adenosine blockade may restore T-cell and NK-cell activity; tryptophan-metabolic reprogramming may disrupt kynurenine-, indole-, serotonin-, or Siglec-15-associated immune escape; and nucleotide-metabolite targeting may weaken macrophage-mediated suppression. The central challenge is to match the dominant metabolic checkpoint to the immune contexture of each tumor. In the future, metabolomics, single-cell profiling, spatial transcriptomics, and spatial metabolite imaging may help identify which metabolic checkpoint should be targeted in which patient, enabling more precise combinations of metabolic therapy and immune checkpoint blockade.

### 6.6 Clinical biomarkers, trial evidence, toxicity, and resistance patterns

For clinical translation, metabolic checkpoint blockade should be guided by biomarker-defined immune-metabolic states. Candidate biomarkers include tumor or plasma lactate, LDHA/MCT expression, lactylation marks, CD39/CD73 activity, adenosine-related signatures, IDO1/TDO expression, kynurenine-to-tryptophan ratios, AhR activation, Siglec-15 expression, and TAM-associated nucleotide-sensing programs (21, 88, 89). However, most remain exploratory and require validation in paired tumor biopsies, blood samples, spatial datasets, and ICB-treated clinical cohorts (88). Clinical evidence is currently strongest for adenosine and tryptophan pathways. Oleclumab and quemliclustat have advanced CD73 blockade into clinical testing, but results suggest that adenosine inhibition requires biomarker-guided patient selection rather than empirical combination with PD-1/PD-L1 blockade (86, 88). The negative ECHO-301/KEYNOTE-252 trial of epacadostat plus pembrolizumab further indicates that tryptophan metabolism cannot be targeted effectively without confirming pathway dependence (77). Lactate-directed therapies, including LDHA, MCT, GPR81, and lactylation-targeted approaches, remain less clinically mature and are supported mainly by preclinical evidence. Potential toxicities should also be considered because lactate, adenosine, and tryptophan metabolism are required for normal immune and tissue homeostasis (89, 94, 95). Broad inhibition may disrupt T-cell activation, macrophage function, vascular regulation, tissue repair, and systemic immune balance. Patient selection should therefore match dominant metabolic features: lactate-high/Treg-rich tumors for lactate-directed therapy, CD73-high/hypoxic immune-excluded tumors for adenosine blockade, kynurenine-high/AhR-active tumors for tryptophan targeting, and TAM-dominant tumors for nucleotide-metabolite intervention. Finally, primary resistance and acquired resistance should be distinguished. Primary resistance may reflect pre-existing metabolic suppression, whereas acquired resistance may arise from therapy-induced metabolic rewiring, requiring longitudinal metabolic and spatial immune monitoring.

## 7 Conclusions and future perspectives

The studies discussed in this mini review support an emerging view that tumor-derived metabolites are not merely nutritional by-products or passive indicators of altered cancer metabolism. Instead, they can function as active immunoregulatory signals that shape the composition, localization, and functional state of immune cells within the tumor microenvironment. Similar to classical immune checkpoints, metabolic immune checkpoints restrict antitumor immunity and contribute to resistance to immune checkpoint blockade. However, unlike receptor-centered checkpoints such as PD-1, PD-L1, or CTLA-4, metabolic checkpoints often act through broader ecological mechanisms, including nutrient competition, metabolite accumulation, extracellular enzyme cascades, metabolite-sensing receptors, post-translational modifications, and myeloid-cell education. Among these pathways, lactate, adenosine, tryptophan-derived metabolites, and nucleotide-derived metabolites represent four major categories of metabolic immune checkpoints. Lactate links tumor glycolysis to immune suppression by promoting Treg recruitment, impairing effector T-cell function, and inducing lactylation-dependent regulatory programs. The lactate/GPR81/CX3CL1 axis, lactate-rich Treg-attracting niches driven by tumor-intrinsic alterations, and lactylation-mediated immune resistance together demonstrate that lactate is not simply a waste product of aerobic glycolysis but a dominant organizer of immune escape. Adenosine represents an enzymatically generated metabolic checkpoint. Through the CD39/CD73 cascade and A2A/A2B receptor signaling, adenosine suppresses CD8^+^ T cells and NK cells while supporting Treg and myeloid-mediated immunosuppression. The CD73-adenosine axis is particularly relevant in immune-excluded, hypoxic, inflamed, and myeloid-rich tumors.

Tryptophan metabolism further illustrates how amino acid-derived metabolites can generate immune tolerance. Beyond the classical IDO1–kynurenine–AhR pathway, recent studies suggest that kynurenine may cooperate with non-classical checkpoint molecules such as Siglec-15, while broader kynurenine/indole and serotonin branches can also influence ICB responsiveness. These findings indicate that tryptophan metabolism should be viewed as a flexible immunoregulatory network rather than a single linear pathway. More recently, nucleotide-derived metabolites have expanded the concept of metabolic immune checkpoints. UDP-driven tumor–macrophage crosstalk shows that cancer cell nucleotide metabolism can reshape TAM states and promote immunotherapy resistance, highlighting the importance of metabolite-mediated myeloid checkpoints. Despite these advances, several important questions remain. First, the spatial distribution of metabolic checkpoints within tumors is still insufficiently defined. Metabolites are not uniformly distributed; they accumulate in hypoxic cores, necrotic regions, stromal barriers, perivascular niches, and myeloid-rich areas. Therefore, future studies should integrate single-cell sequencing, spatial transcriptomics, spatial proteomics, metabolomics, and spatial metabolite imaging to determine where specific metabolic checkpoints operate and which immune cells they affect. Second, association does not necessarily prove causality. Functional perturbation in organoids, mouse models, patient-derived models, and clinical samples will be necessary to validate whether targeting a given metabolic pathway can truly restore antitumor immunity. Third, metabolic pathways are highly context-dependent. The same metabolite may have different effects depending on tumor type, immune composition, nutrient availability, microbiota, stromal architecture, and treatment pressure.

Future research should move from general association studies toward actionable immune-metabolic mapping. Several specific questions need to be addressed. First, which metabolic checkpoints dominate in different immune-resistant niches, such as lactate-rich Treg-enriched regions, CD73-high hypoxic immune-excluded regions, kynurenine/AhR-active suppressive niches, or TAM-dominant UDP-responsive areas? This question can be addressed by integrating spatial metabolomics, single-cell RNA sequencing, multiplex immunofluorescence, imaging mass spectrometry, and paired pretreatment/on-treatment tumor biopsies. Second, which metabolic checkpoints are drivers rather than passengers of ICB resistance? This requires perturbation-based validation using CRISPR screening, isotope tracing, organoid–immune cell co-culture systems, patient-derived xenografts, and syngeneic immunotherapy models. Third, which biomarkers can guide patient selection? Candidate biomarker panels should combine metabolite levels, enzyme expression, receptor activation, lactylation marks, immune-cell composition, and clinical response data. Fourth, future drug development should prioritize dual-target or network-targeted strategies, such as LDHA/CD73, MCT/A2AR, IDO1/AhR, or metabolic checkpoint blockade combined with macrophage reprogramming. Finally, prospective clinical trials should stratify patients according to dominant immune-metabolic phenotypes and distinguish primary metabolic resistance from acquired metabolic rewiring after ICB exposure.

Therapeutically, the major challenge is to match the correct metabolic intervention with the dominant immune resistance state. Lactate-directed strategies may be most useful in glycolytic, Treg-rich tumors; CD73-adenosine blockade may benefit immune-excluded or hypoxic tumors; tryptophan-metabolic reprogramming may be relevant for tumors with kynurenine-, AhR-, or Siglec-15-associated immune escape; and nucleotide-metabolite targeting may be valuable in TAM-dominant resistant niches. Together, these studies support a conceptual shift from receptor-centered immune checkpoints to metabolite-centered immune checkpoints. Defining where, when, and how tumor-derived metabolites suppress antitumor immunity may provide new opportunities to overcome resistance to immune checkpoint blockade.

The novelty of the metabolic immune checkpoint concept lies not in describing individual metabolites separately, but in recognizing that tumor-derived metabolites form a multilayer immune-regulatory network. In this mini review, we classify these pathways into accumulation, enzymatic conversion, receptor-sensing, epigenetic-imprinting, and myeloid-education checkpoints. This framework distinguishes metabolite-centered immune suppression from classical receptor-centered checkpoints and may help guide biomarker-driven combinations of metabolic intervention with immune checkpoint blockade.

Future studies should move from single-metabolite analysis toward network-level immune-metabolic mapping. Lactate, adenosine, tryptophan-derived metabolites, UDP signals, lipid mediators, ROS, arginine depletion, and glutamine rewiring may cooperate to create spatially organized immune-resistant niches. Defining these crosstalk patterns will be essential for selecting rational combinations, such as LDHA or MCT blockade with CD73/A2A inhibition, tryptophan-metabolic reprogramming with checkpoint blockade, or myeloid-directed metabolic therapy in TAM-dominant tumors.

Overall, the next stage of metabolic checkpoint research should focus on three translational goals: detecting spatially restricted metabolic suppression, validating causal metabolic drivers of immune resistance, and designing biomarker-guided combination regimens. By linking spatial detection technologies with functional perturbation models and rational dual-target drug development, metabolic immune checkpoint research may move from descriptive pathway analysis toward clinically actionable strategies for overcoming immunotherapy resistance.
